# Evaluation of Exposure to Toluene and Xylene in Gasoline Station Workers

**DOI:** 10.1155/2021/5553633

**Published:** 2021-05-20

**Authors:** Barbara R. Geraldino, Rafaella F. N. Nunes, Juliana B. Gomes, Katia S. da Poça, Isabela Giardini, Paula V. B. Silva, Helen P. Souza, Ubirani B. Otero, Marcia Sarpa

**Affiliations:** ^1^Technical Area of Environment, Work and Cancer, Prevention and Surveillance Coordination, National Cancer Institute José Alencar Gomes da Silva (INCA), Rua Marquês de Pombal 125, 5º Andar-Centro, Rio de Janeiro CEP 20230-240, Brazil; ^2^Environmental Mutagenesis Laboratory, Department of Biochemistry, Biomedical Institute, Federal University of Rio de Janeiro State (UNIRIO), Rua Frei Caneca 94, 4º Andar-Centro, Rio de Janeiro CEP 20211-040, Brazil

## Abstract

The main volatile organic compounds found at gasoline stations are benzene, toluene, ethylbenzene, and xylene isomers (BTEX). They cause several harmful effects on human health. Regulatory Norm 7 (1978) provides that, in Brazil, biological monitoring of toluene and xylene is carried out by measuring the urinary metabolites hippuric acid (HA) and methylhippuric acid (MHA), respectively. The objective of this study was to assess the exposure to toluene and xylene and to identify related signs and symptoms in gasoline station workers. A cross-sectional epidemiological study was conducted with workers occupationally exposed to fuels. These gasoline station workers were divided into two groups: 94 workers exposed mainly by inhalation (convenience store workers (CSWs)) and 181 workers exposed by inhalation and dermal route (filling station attendants (FSAs)). A comparison group was formed by 119 workers not occupationally exposed to fuels (office workers (OWs)). Workers exposed to fuels had higher average levels of these exposure biomarkers (HA and MHA), which were also higher in convenience store workers than in filling station attendants. In addition, individuals exposed to the solvents present in gasoline had altered mood/depression, cramps, dizziness, drowsiness, headaches, irritability/nervousness, weakness, weight loss, and other symptoms more frequently and had higher urinary levels of HA and MHA compared to the comparison group. Gasoline station workers showed high levels of HA and MHA, reflecting high occupational exposure to the solvents toluene and xylene present in gasoline, demonstrating that changes in the current legislation and in the work environment are necessary to ensure better health protection for these workers.

## 1. Introduction

Gasoline station is establishments that retail liquid fuels derived from petroleum, alcohol, and other automotive fuels, with equipment available for their measurement and storage [1]. This process, associated with gasoline distribution, can be an important source for emission of volatile organic compounds (VOCs) [2], with benzene, toluene, ethylbenzene, and xylene isomers (BTEX), the main volatile hydrocarbons originating from petroleum. The intense use of fuels added to the high evaporation rates is the main factor of atmospheric pollution, especially in occupational environments such as gasoline stations, due to the proximity of the emission source [3, 4]

VOCs are absorbed mainly through the respiratory and dermal routes. At least 80% of these compounds are metabolized in the body and excreted by urine. Only a small fraction is excreted unchanged in the exhaled air [5]. Benzene is classified by the International Agency for Research on Cancer (IARC) as a carcinogen for humans (Group 1), associated with the development of chronic myeloid leukemia, multiple myeloma, acute myeloid leukemia in children, and lung cancer [6]. Ethylbenzene is classified as possibly carcinogenic to humans (Group 2B), while toluene and xylene have so far had poor evidence of carcinogenicity (Group 3) [7]. These classifications are not static, and agents may change their classification as new studies present sufficient evidence for possible changes.

Although there is still no evidence for the development of cancer due to toluene, acute exposure causes immediate excitability and euphoria, followed by a depressing response with disorientation, mood fluctuations, hallucinations, and ataxia [8, 9]. Toluene is known to be neurotoxic and some of the chronic effects observed after prolonged periods of exposure include memory/concentration problems, disturbance of emotional and psychomotor functions, hepatotoxicity, nephrotoxicity, and hearing loss, which can cause permanent brain damage or even lead to death [9–11].

In turn, acute exposure to xylene can cause nausea, headache, and vomiting; furthermore, prolonged contact with the skin can cause irritation and dermatitis [12, 13]. Chronic exposure to xylene can cause liver and kidney damage, with increased blood urea levels, pulmonary congestion, respiratory failure, and hepatomegaly [9]. Teratogenic effects can also be observed [14], affecting the development of embryos and fetuses during maternal exposure. Moreover, studies report that xylene and toluene can interfere with fertility, affecting semen quality [15], and the central nervous system, leading to a slow response to external stimuli [16], memory impairment, body gait imbalance, and lack of motor coordination, among other outcomes [9].

The greatest exposure to these agents occurs in the work environment. In addition to gasoline station workers (GSWs), those in industries, such as chemical, petroleum, rubber, and the production and use of paints, solvents, varnishes, and adhesives, are among the categories most cited for greater exposure to toluene and xylene [9]. Benzene, toluene, and xylene are added to gasoline to improve its octane rating, resistance to detonation index [9]. Thus, GSWs are among those with the highest exposure and, in Brazil, due to the nature of the work process that includes daily and direct contact with fuels, especially gasoline [17], the risks arising from exposure may be increased.

Inhalation is the main route of exposure to toluene and xylene in the body. After absorption, these VOCs undergo metabolic transformation processes, involving cytochrome P450 enzymes in the liver, such as CYP2E1, which lead to the formation of various metabolites excreted in the urine [18, 19]. The main metabolites of toluene are ortho-cresol and benzaldehyde intermediate, which is conjugated with glycine and forms hippuric acid [14]. In the case of xylene, the metabolite produced after conjugation with glycine is methylhippuric acid, which can be in the form of three isomers: para-, meta-, and ortho-methylhippuric [20].

The urinary metabolites hippuric acid (HA) and methylhippuric acid (MHA) are used as biomarkers of exposure to toluene and xylene, respectively, and are used to monitor and quantify worker's exposure levels. In Brazil, the parameters for biological control of occupational exposure to toluene and xylene are established by Regulatory Norm 7 (NR7), which is the current legislation since 1978 [21]. The reference value according to NR7 is 1.5 g/g of creatinine for HA. This value is expected to be found in people who are not occupationally exposed, but the biological exposure index (BEI) is 2.5 g/g of creatinine because these metabolites, after a day of exposure, can accumulate in adipose tissue [9]. For MHA, the BEI is 1.5 g/g of creatinine, with no reference value, since this metabolite is not expected to be found in individuals not occupationally exposed to xylene [21].

Biological monitoring of toxic substances is important to confirm exposure, estimate the levels of the chemical agent or its metabolites in the body (internal dose), and allow detection of early signs of possible damage, in addition to preventing the development and progression of occupationally related diseases [22]. The aim of the present study was to assess exposure to toluene and xylene and to identify related signs and symptoms in GSWs.

## 2. Materials and Methods

### 2.1. Geographic Characteristics of Regions

The present study was carried out at gasoline stations located in the Center and the South Zone of the City of Rio de Janeiro. The central area of Rio de Janeiro is predominantly commercial, consisting of tall buildings, old houses, and population aggregation such as small- and medium-sized communities. This region contains the main roads that connect the city to expressways, which results in intense vehicle traffic. The South Zone region is an area of great real estate appreciation [22], with high infrastructure standards. It has a milder microclimate than the Center of Rio de Janeiro, due to a wide variety of wooded areas, in addition to natural landscapes such as Rodrigo de Freitas Lake, Botanical Garden, and coastal area with numerous beaches.

### 2.2. Study Design and Population

A cross-sectional epidemiological study was carried out with workers from 21 gasoline stations, 9 of which are located in the Central region and 12 in the South Zone of the Municipality of Rio de Janeiro. At these gasoline stations, 384 workers were recruited and interviewed, divided into two groups. The first group (convenience store workers (CSWs)) was formed by administrative workers and convenience store attendants. They did not work directly with the fuel and for this reason were exposed to toluene and xylene in the work environment mainly by inhaling gasoline vapors. The second group (filling station attendants (FSAs)) was composed of workers directly exposed to toluene and xylene, who supply vehicles and are exposed by inhalation of vapors and dermal contact with gasoline (e.g., attendants, managers, submanagers, and lubricators). A control group was also included, for comparison, composed of workers without occupational exposure to fuels (office workers (OWs)). This group included 119 individuals from the administrative areas of the National Cancer Institute (INCA) and the Federal University of the State of Rio de Janeiro (UNIRIO).

Of the 503 eligible individuals, 109 of 384 samples (28.4%) from the exposed group and 19 of 119 samples (16%) from the OW group were excluded from toxicological analyses because they had urinary creatinine levels outside the range (<0.3; >3 g/L) recommended by ACGIH [23]. Thus, the final study population had 375 participants—275 from the exposed group and 100 from the comparison group.

The sampling of gasoline station was nonprobabilistic and, in all establishments, employees over the age of 18 were invited to participate in the study, regardless of their role. Workers with less than six months of work were not included. The study was approved by the INCA Research Ethics Committee (registration number 121/09).

### 2.3. Data Collection

After recruitment, an individual questionnaire with sociodemographic information and signs and symptoms was applied to the participants. The influence of factors such as gender, smoking, food, alcohol, and processed food consumption was investigated, due to the possible interference of some habits in the urinary levels of HA and MHA.

### 2.4. Sample Collection

Urine samples were collected at the end of the day in three work shifts lasting 8 hours each (departure at 6 a.m., 2 p.m., and 10 p.m.). After collection, the urine samples were packed in a polystyrene box with recyclable ice and transported to the laboratory for analysis (HA, MHA, and urinary creatinine). Samples were collected once for each worker and analyzed separately. Samples of OWs (comparison group) were collected at the end of the working day and handled in a similar way as the exposed group (GSWs: CSW and FSA).

### 2.5. Urinary Creatinine

Measurements of urinary creatinine levels were performed up to 12 hours after collection and determined by the colorimetric endpoint (Bioclin®) [24]. All urine samples that showed creatinine levels less than 0.3 g/L or greater than 3.0 g/L were excluded from the analysis, following the guidelines of the American Conference of Governmental Industrial Hygienists [23].

### 2.6. HA and MHA

The urinary levels of HA and MHA were evaluated by high-performance liquid chromatography (HPLC) using an ultraviolet detector (HPLC-UV) equipped with a Lichrosorb RP18 chromatography column (244 × 4 mm) with Merck 5 mm particles® [25]. The standard was obtained from Sigma-Aldrich®, and the normalization of the results was adjusted by the urinary creatinine levels. 1 mL of sample was added to 1 mL of methanol and then centrifuged for 7 min at 3000 rpm, with subsequent manual injection of 20 µL into the HPLC-UV.

### 2.7. Chromatographic Conditions for HA Quantification

The mobile phase consisted of an aqueous solution of methanol and concentrated acetic acid (792 : 200 : 8 v/v) at a flow rate of 1.3 mL/min. The total chromatographic run time was 12 min; the detector was kept at *λ* = 257 nm for reading and the column at 40°C. The detection limit was 0.0075 mg/L.

### 2.8. Chromatographic Conditions for MHA Quantification

The mobile phase consisted of a mixture of 50 mM phosphate buffer and methanol (7 : 3 v/v) at a flow rate of 1.6 mL/min. The total time of the chromatographic run was 8 min; the detector was kept at *λ* = 238 nm for reading; and the column was maintained at 40°C. The detection limit was 0.005 mg/L.

### 2.9. Statistical Analysis

Initially, descriptive statistics were performed for the following variables: sex, tobacco consumption, alcoholic beverages, processed food consumption, and length of stay in the current job. The Kolmogorov–Smirnov test was used to verify the normality in the distribution of urinary levels of HA and MHA in the samples. After that, measures of central tendency (mean and median) and 95th percentile of HA and MHA were presented, stratified between OWs (comparison group), CSWs (exposed mainly by respiratory route), and FSAs (exposed mainly by respiratory and dermal routes). The difference between the categories was performed using the Kruskal–Wallis test.

To better understand the distribution of the HA and MHA values of the study population, we decided to categorize the median values of the biomarkers according to the average values of the OW. For hippuric acid, the value used as the cutoff point was ≤ 0.083. For methylhippuric acid, the value was ≤ 0.016. The difference between the categories was performed using the chi-square test.

We assessed whether possible signs and symptoms were related to exposure to the toluene and xylene hydrocarbons in the workplace. The symptoms asked in a questionnaire were altered mood/depression, anxiety, asthenia, attention deficit/hyperactivity disorder, cramp, difficulty seeing, dizziness, drowsiness, headache, insomnia, involuntary movement, irritability/nervousness, memory loss, seizure, tingling, tremor, weakness, and weight loss. The difference between the categories was performed using the chi-square test and Kruskal–Wallis test with Dunn's posttest.

Statistical analyses were performed using the Statistical Package for the Social Sciences (SPSS) for Windows version 17.0, and figures were produced by GraphPad Prism.

## 3. Results

The sociodemographic characteristics of the studied population are shown in Table 1. The OW and CSW groups were mostly female, while the FSA group was mostly male. The average age of the FSAs (37 years old) and OWs (39) was a similar age, but the CSWs were younger at only 29 years old. Most workers in the 3 study groups reported not using tobacco. However, the consumption of alcoholic beverages was reported in all groups as well as daily consumption of industrialized food. All groups reported practicing the profession for up to 9 years.

As presented in Table 2, the workers in the exposed group had higher average urinary levels of HA (g/g creatinine), at about 4.7 (CSWs) and 3.8 (FSAs) times higher, than the comparison group (OWs). As for urinary MHA levels (g/g creatinine), the GSWs presented an average 100 times higher than the OW group. In addition, most of the CSW (92.6%) and FSA (86.2%) had values of HA higher than 0.083 g/g creatinine as shown in Table 2. When the baseline reference value adopted was the detection limit, these frequencies were even higher (CSW: 98.9% and FSA: 95.6%) (data not shown). For xylene, 98.9% of CSWs and FSAs had much higher MHA values than the adopted reference category (0.016 g/g creatinine).

**Figure 1:**
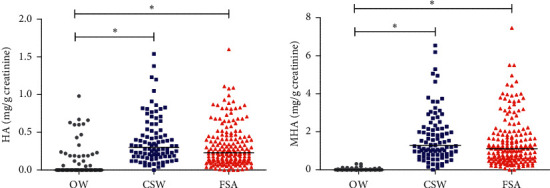
Scatter chart for values of hippuric acid and methylhippuric acid of gasoline station workers (occupationally exposed to VOCs) and office workers (comparison group) in Rio de Janeiro (Brazil), 2015–2017. Kruskal–Wallis test, *p* value < 0.001, with Dunn's posttest, *p* value < 0.05.

Comparing the values of HA and MHA in urine, the exposed workers had similar levels at biomarkers, although GSWs had the highest values in measurements of central tendency. Furthermore, both exposed groups (CSW and FSA) had both biomarkers higher than the comparison group (OW), with a statistically significant difference. This difference was not observed between the CSW and FSW groups (Figure 1).

Unexpectedly, HA levels in individuals who reported not consuming processed foods had a higher median (0.280 g/g creatinine) than in those who claimed to consume them seldom (0.065 g/g creatinine), 1-2 times a week (0.215 g/g creatinine), 3–6 times a week (0.190 g/g creatinine), and daily (0.185 g/g creatinine), with a statistically significant difference (*p* value < 0.05). In contrast, individuals who self-reported consuming processed foods 1-2 times a week had a higher median (0.910 g/g creatinine) of MHA compared to those who claimed to consume them seldom (0.320 g/g creatinine), 3–6 times a week (0.870 g/g creatinine), daily (0.785 g/g creatinine), and nonconsuming (0.470 g/g creatinine), with no statistically significant difference (*p* value = 0.096) (data not shown).

Table 3 exhibits the frequency and median values (g/g creatinine) of the HA and MHA biomarkers of the participants who answered Yes for the different signs and symptoms. Workers exposed to toluene and xylenes present in gasoline (CSWs and FSAs) obtained the highest medians for the HA and MHA biomarkers with a statistical difference (*p* < 0.05 < 0.05). Furthermore, the majority of those who answered Yes were also part of the GSW group. However, only altered mood/depression, cramp, dizziness, drowsiness, weakness, and weight loss were more frequent in the exposed group with statistical difference (*p* < 0.05). Only drowsiness and weakness were more frequent in both exposed groups (CSW and FSA).

When the values of HA and MHA biomarkers were evaluated among those exposed, a difference in the cramp, drowsiness, and headache for the HA biomarker could be observed from CSW and FSA groups. The HA values (g/g creatinine) were higher for the CSW group (Table 3). For the MHA biomarker, only cramp and irritability/nervousness were higher for GSWs. However, CSW groups had higher MHA values for cramp, and the FSA group had higher MHA values for irritability/nervousness (Table 3). These differences in signs and symptoms were statistically significant (*p* < 0.05). Two workers in the CSW group and one worker in the FSA group reported seizures, which were not reported by anyone in the comparison group, OW (data not shown).

## 4. Discussion

The present study showed a higher concentration of HA and MHA in the urinary levels of GSWs, who were exposed to solvents present in gasoline when compared to OWs (comparison group). The sociodemographic characteristics prevalent in the exposed group are similar to other studies carried out with GSWs. Campos [26] conducted a study with 23 workers at gasoline station in Recife City, Brazil, where the majority were male (78.3%) and had worked for less than 10 years at this profession (95.7%). Another study carried out by Rocha [27], with 221 workers in the same category, also found that the majority of the studied population was male (90.5%) and had held the job for less than 10 years (81.4%), data consistent with the findings in the present study [26, 27].

The mean and median HA urinary levels found in the groups occupationally exposed to toluene (CSWs and FSAs) were lower than those permitted for BEI and established by the NR7 of 2.5 g/g of creatinine [21]. However, this does not mean that the daily exposure cannot affect their health. In this study, the average urinary HA levels of the OW group (comparison group) were substantially lower than in the GSWs. When using the average HA values of the control group (as a cutoff point), more than 85% of GSWs had these urinary values greater than 0.083 g/g creatinine.

Pelclová and collaborators [28], evaluating printing workers, found that HA levels were 3 times higher among those exposed than in the control group, and the percentages of aberrant cells were 2.30 in the printers and 1.46 in the control group (*p* < 0.05) [28]. Findings like this demonstrate the inadequacy of the high values of the biomarker referenced by the current legislation, considering that workers not exposed to solvents had levels much lower than the reference value, which is 1.5 g/g creatinine. The establishment of high levels allows workers to be exposed daily to higher concentrations of solvents in the air, without adopting protective measures. Situations like these result in unhealthy working conditions.

For MHA levels, xylene is present only in those occupationally exposed, and their urinary metabolite levels were even more worrying. Although the means and medians in the exposed group were also lower than the BEI established by the NR7 of 1.5 g/g of creatinine [21], when comparing the GSW group exposed to xylene with OWs not exposed, the average was about 100 times greater, with a statistically significant difference. This indicates that GSWs are exposed to a high concentration of this hydrocarbon and consequently to the harmful effects caused by xylene. Geraldino and collaborators [29] with the same study population assessed benzene exposure levels and showed that the FSA had average values about twice as high of *t*, t-MA (0.219 mg/g creatinine; 95% CI 0.174–0.264) compared to OW (0.126 mg/g creatinine; 95% CI 0.0817–0.1693). The levels of *T*, t-MA were higher in workers of CSWs on gasoline only by inhalation (0.267 mg/g creatinine; 95% CI 0.157–0.376) than those exposed to gasoline by inhalation and dermal, attending gas stations (0.195 mg/g creatinine; 95% CI 0.155–0.235) [29].

Our study found that, among those exposed to the solvents toluene and xylene present in gasoline, the levels of HA and MHA were almost all higher among CSWs (exposed mainly through the respiratory route) than in the FSA group (exposed mainly through the respiratory and dermal). Differences in working conditions and environments for these groups may justify this variation since the VOCs evaluated have high volatility as their main characteristic. These vapors remain at low heights, which facilitates airway penetration [30].

Another important factor is that while the FSAs spend most of their time in large and airy places, the CSW group generally works in closed environments with low air circulation. This low air circulating is suitable for longer confinement of toluene and xylene at the workplace. Literature data discuss this condition, as in the example of Lebret and collaborators [31], who assessed VOC levels within more than 300 households in the Netherlands. The authors observed an 8 times higher concentration of these solvents indoors than outdoors [31]. Schneider and collaborators [29] also observed higher levels of BTXs in indoor areas, compared to outdoor areas, during a study carried out in Germany [32].

The levels of HA present a good correlation with the levels of exposure to toluene [33]. However, it may suffer interference with some chemical agents; therefore, HA is not considered a specific biomarker. Although several studies demonstrate the influence of alcohol and tobacco consumption on HA levels [34, 35], since toluene is part of the composition of cigarettes [36], no significant differences were observed in the urinary excretion of this metabolite in relation to alcohol and tobacco consumption, in the GSW group. This finding agrees with other studies that demonstrated that these factors did not significantly alter baseline levels of the HA biomarker [33, 37].

Even individuals not exposed to aromatic organic solvents can present this metabolite in their urine. A diet rich in foods that contain the preservative benzoic acid and/or its precursors increases the level of HA in the urine [38]. The data presented here did not show this increase, since the highest levels of urinary HA were present in workers who reported not consuming processed foods. However, the difficulty in defining processed foods by the interviewees may have interfered with the result obtained. This does not guarantee that the participant's diet is free of preservatives since a wide variety of processed and natural foods contain benzoic acid in their formulation [39].

Some studies point out that, after a person ingests large amounts of liquids, the concentration of a substance or its metabolites in their urine decreases [40–42]. However, we observed when the interviews were being conducted and biological samples were being collected that, among the GSWs, the work routine of the interviewed FSAs hinders frequent departures for drinking water and using the bathroom. The same is not generally the case with office and administration workers who find it easier to drink liquids during the day.

Some samples were excluded to not influence the results. This occurred due to the impossibility of collecting new urine samples from workers with creatinine levels outside the acceptable limits (< 0.3 g/L or > 3.0 g/L) [23]. The highest percentage of workers excluded from this study was from the GSWs, which can be a limitation. It is known that the production and excretion of creatinine are considered constant and comparable for healthy individuals. This allows creatinine to be used as an estimate of the dilution or concentration of urine [43, 44].

Some studies suggest that occupational exposure to toluene and xylene is related to a higher incidence of acute and chronic signals and symptoms. Among the signs and symptoms evaluated here, altered mood/depression, cramps, dizziness, drowsiness, weakness, and weight loss were shown to be more related to exposure to toluene (HA) and xylene (MHA) due to the higher frequency of Yes respondents and/or higher urinary values of the HA and MHA biomarkers. In this study, there was also a greater tendency for headaches with exposure to toluene (HA levels) and irritability/nervousness with exposure to xylene (MHA levels). In addition to these, the other signs and symptoms assessed were identified as possible changes resulting from exposure to toluene and/or xylene. The other signs and symptoms identified were anxiety, asthenia, attention deficit/hyperactivity disorder, difficulty seeing, insomnia, involuntary movement, memory loss, seizure, tingling, and tremor.

The literature reports acute and chronic exposure to toluene causes irritation eyes, nose, lassitude (weakness; exhaustion), confusion, excitability and euphoria, dizziness, headaches, dilated pupils, lacrimation, anxiety, muscle fatigue, insomnia, paresthesia, depressing response with disorientation, mood fluctuations, hallucinations, and ataxia, hearing loss, dermatitis, liver and kidney damage, deficits in memory/concentration, emotional disturbance, and psychomotor problems [8–11, 45].

In the case of occupational exposure to xylene, headaches, dizziness, ataxia, drowsiness, excitement, tremor, nausea, vomiting, irritation, and dermatitis are some symptoms observed [12, 13, 46]. Interference in the central nervous system with a slow response to external stimuli, memory impairment, body gait imbalance, and lack of motor coordination has also been reported for exposure to xylene and toluene [9, 16].

It is important to note that toluene is a widely used solvent, but there are limited human data in the literature with exclusive exposure to this substance. Its association, in commercial preparations, with other substances, such as xylene and benzene in gasoline, makes it difficult to identify pathologies and even specific signs and symptoms [47].

For biomonitoring occupational exposure to toluene, ACGHI determines the measurement of the urine ortho-cresol metabolite (BEI 0.3 mg/g creatinine) or urine toluene (BEI 0.03 mg/L) at the end of the work shift. Blood toluene (BEI 0.02 mg/L) can also be used before the last working day of the week. However, ortho-cresol may be influenced by passive or active smoking. Nevertheless, o-cresol never exceeds that indicated in the BEI. Measurement of urine toluene should be performed to confirm exposure above 100 ppm in the workplace, which is an orientation followed by many countries like New Zealand and Australia [48], where xylene is measured by urinary MHA (BEI 1.5 g/g creatinine) collected at the end of work shift [49].

In Brazil, it is extremely important to emphasize that Regulatory Norm 7 (NR7) of the Brazilian Ministry of Labor [21] was updated. Ordinance No. 6,734 of March 9, 2020 [50], will be effective beginning in March 2021. This regulation provides an updated chart containing a list of some chemical agents that have biological indicators to be analyzed periodically to better monitor workers for exposure to certain chemical agents.

The original standard provided the use of urinary HA exposure biomarker, for the assessment of workers' exposure to toluene [21]. The revision of the standard provides for the HA biomarker to be replaced by measuring the toluene itself in the blood (0.02 mg/L) and/or urine (0.03 mg/L) or the presence of ortho-cresol in the urine (0.3 mg/g creatinine), in line with international standards [48]. For the biomonitoring of xylene, in the current version of NR7, the acceptable value will go from 1.5 g/g of creatinine to 1.5 mg/g of creatinine [48], making the legislation much more restrictive.

The new legislation will use new biomarkers, and it appears that it will provide greater protection to workers. However, the results presented here show that the current occupational exposure to toluene and xylene leads to much higher HA and MHA values (g/g creatinine) among those exposed. Related to these high values of HA and MHA, all the signs and symptoms evaluated were higher among those exposed. The frequency of exposed workers who reported signs and signals include the presence of altered mood/depression, cramps, dizziness, drowsiness, weakness, and weight loss, which all exhibited statistically significant differences.

## 5. Conclusions

This study demonstrated that urinary HA and MHA levels were higher in the group of workers occupationally exposed to toluene and xylene (GSWs) than the unexposed group (OWs). Furthermore, there was the observation of altered mood/depression, cramps, dizziness, drowsiness, headaches, irritability/nervousness, weakness, weight loss, and other symptoms for exposed workers who responded Yes for some signs and symptoms or had higher levels of urinary HA or MHA.

The change in national legislation for the biomonitoring of exposure to toluene can provide more accurate information about the real levels of workers' exposure to toluene because the internal dose can be determined with greater specificity. This legislation also changed the value limits for MHA, a biomarker for xylene. Such findings are important, as they help to understand the levels of exposure to solvents, prevent excessive exposure, and promote effective changes in work processes. These changes can minimize the risks and damages to worker's health and improve the quality of life and work of these individuals.

## Figures and Tables

**Table 1:** Descriptive analysis of the sociodemographic characteristics of gasoline station workers (occupationally exposed to VOCs) and office workers (comparison group) in Rio de Janeiro (Brazil), 2015–2017.

Variables	Office workers^1^ (*n* = 100)	Gasoline station workers^2^ (*n* = 275)	
		Convenience store workers (*n* = 94)	Filling station attendants (*n* = 181)
	*n* (%)	*n* (%)	*n* (%)
Sex			
Male	46 (46.0)	28 (29.8)	161 (89.0)
Female	54 (54.0)	66 (70.2)	20 (11.0)

Age			
Median (min; max)	39 (20; 60)	29 (20; 67)	37 (20; 70)

Smoking			
Nonsmoker	86 (86.0)	72 (76.6)	115 (63.5)
Ex-smoker	8 (8.0)	15 (16.0)	31 (17.1)
Smoker	6 (6.0)	7 (7.4)	35 (19.3)

Alcohol consumption			
No	34 (34.0)	40 (42.6)	58 (32.0)
Yes	66 (66.0)	54 (57.4)	123 (68.0)

Processed food consumption			
No	8 (8.0)	4 (4.3)	11 (6.1)
Rarely	9 (9.0)	3 (3.2)	10 (5.5)
1-2 times a week	20 (20.0)	29 (30.9)	59 (32.6)
3–6 times a week	14 (14.0)	20 (21.3)	37 (20.4)
Daily	48 (48.0)	37 (39.4)	61 (33.7)
Could not answer	1 (1.0)	1 (1.1)	3 (1.7)

Working time (years)			
≤9	57 (57.0)	83 (88.3)	136 (75.1)
9 < *X* ≤ 20	13 (13.0)	9 (9.6)	21 (11.6)
>20	5 (5.0)	0 (0.0)	24 (13.3)
Could not answer	25 (25.0)	2 (2.1)	0 (0.0)

^1^Not occupationally exposed to toluene and xylene. ^2^Occupationally exposed to toluene and xylene.

**Table 2:** Mean, P95 values, and distribution of workers by the levels of hippuric acid and methylhippuric acid in gasoline station workers (occupationally exposed to VOCs) and office workers (comparison group) from Rio de Janeiro (Brazil), 2015–2017.

Variables	Office workers^1^ (*n* = 100)	Gasoline station workers^2^ (*n* = 275)		*p* value
		Convenience store workers (*n* = 94)	Filling station attendants (*n* = 181)	
Toluene (hippuric acid (HA))				
Mean ± SD	0.083 ± 0.19	0.393 ± 0.308	0.319 ± 0.271	0.001^*∗*^
P95	0.609	1.087	0.869	
Reference: mean of OW–n (%)^3^				
≤0.083	79 (79.0)	7 (7.4)	25 (13.8)	<0.001^#^
>0.083	21 (21.0)	87 (92.6)	156 (86.2)	
Xylene (methylhippuric acid (MHA))				
Mean ± SD	0.016 ± 0.051	1.714 ± 1.333	1.456 ± 1.266	0.001^*∗*^
P95	0.110	4.977	3.996	
Reference: means of OW–n (%)				
≤0.016	85 (85.0)	1 (1.1)	2 (1.1)	<0.001^#^
>0.016	15 (15.0)	93 (98.9)	179 (98.9)	

^1^Not occupationally exposed to toluene and xylene. ^2^Occupationally exposed to toluene and xylene. ^3^Mean (g/g creatinine) of the office worker group was used as a cutoff point. ^*∗*^Kruskal–Wallis test. ^#^Chi-square test.

**Table 3:** Distribution and values of HA and MHA biomarkers on signs and symptoms of gasoline station workers (occupationally exposed to VOCs) and office workers (comparison group) in Rio de Janeiro (Brazil), 2015–2017.

Variables	Office workers^1^ (*n* = 100)		Gasoline station workers^2^ (*n* = 275)				*p* value^3^
			Convenience store workers (*n* = 94)		Filling station attendants (*n* = 181)		
	*n* (%)	Median (min; max)	*n* (%)	Median (min; max)	*n* (%)	Median (min; max)	
Altered mood/depression							
HA	20 (20.0)	0.00 (0; 0.67)	39 (41.5)^*∗*^	0.32 (0.06; 0.86)	44 (24.3)	0.22 (0; 1.60)	<0.0001
MHA	20 (20.0)	0.00 (0; 0.30)	39 (41.5)^*∗*^	1.31 (0.22; 3.80)	44 (24.3)	1.25 (0; 7.46)	

Anxiety							
HA	57 (57.0)	0.00 (0; 0.67)	51 (54.2)	0.32 (0; 10.38)	84 (46.4)	0.20 (0; 1.60)	<0.0001
MHA	57 (57.0)	0.00 (0; 0.31)	51 (54.2)	1.31 (0; 6.19)	84 (46.4)	0.87 (0; 7.46)	

Asthenia							
HA	14 (14.0)	0.00 (0; 0.98)	11 (11.7)	0.28 (0.08; 0.71)	35 (19.3)	0.30 (0; 1.60)	0.0006
MHA	14 (14.0)	0.00 (0; 0.03)	11 (11.7)	1.05 (0.29; 2.92)	35 (19.3)	1.31 (0; 7.46)	<0.0001

Attention deficit/hyperactivity disorder							
HA	27 (27.0)	0.00 (0; 0.67)	24 (25.5)	0.28 (0.06; 1.05)	42 (23.2)	0.29 (0; 1.11)	<0.0001
MHA	27 (27.0)	0.00 (0; 0.31)	24 (25.5)	1.06 (0.29; 4.95)	42 (23.2)	1.42 (0; 5.50)	

Cramp							
HA	23 (23.0)	0.00 (0; 0.98)	47 (50.5)^*∗*^	0.35 (0.04; 1.38)^#^	59 (32.6)	0.22 (0; 1.08)	<0.0001
MHA	23 (23.0)	0.00 (0; 0.31)	47 (50.5)^*∗*^	1.46 (0.16; 6.19)^#^	59 (32.6)	1.10 (0; 4.43)	

Difficulty seeing							
HA	34 (34.0)	0.00 (0; 0.60)	26 (27.7)	0.29 (0.09; 1.23)	54 (29.8)	0.26 (0; 1.60)	<0.0001
MHA	34 (34.0)	0.00 (0; 0.31)	26 (27.7)	1.27 (0.22; 5.06)	54 (29.8)	1.19 (0; 7.46)	

Dizziness							
HA	12 (12.0)	0.00 (0; 0.19)	40 (42.6)^*∗*^	0.30 (0.04; 1.54)	41 (43.6)	0.21 (0; 1.60)	<0.0001
MHA	12 (12.0)	0.00 (0; 0.09)	40 (42.6)^*∗*^	1.15 (0.16; 6.54)	41 (43.6)	0.88 (0; 7.46)	

Drowsiness							
HA	25 (25.0)	0.00 (0; 0.67)	46 (48.9%)^*∗*^	0.36 (0; 1.54)^#^	76 (42.0%)^*∗*^	0.22 (0; 1.60)	<0.0001
MHA	25 (25.0)	0.00 (0; 0.30)	46 (48.9%)^*∗*^	1.52 (0; 6.54)	76 (42.0%)^*∗*^	1.10 (0; 7.46)	

Headache							
HA	34 (34.0)	0 (0.00; 0.98)	46 (48.9%)	0.28 (0.06; 1.38)^#^	62 (34.2%)	0.22 (0; 1.11)	<0.0001
MHA	34 (34.0)	0.00 (0; 0.30)	46 (48.9%)	1.24 (0.22; 6.19)	62 (34.2%)	1.04 (0; 5.51)	

Insomnia							
HA	24 (24.0)	0.00 (0; 0.47)	28 (29.7)	0.30 (0.06; 0.82)	42 (23.2)	0.24 (0; 1.60)	<0.0001
MHA	24 (24.0)	0.00 (0; 0.31)	28 (29.7)	1.31 (0.31; 3.58)	42 (23.2)	1.15 (0; 7.46)	

Involuntary movement							
HA	15 (15.0)	0.00 (0; 0.43)	27 (28.7)	0.28 (0.06; 1.23)	44 (24.3)	0.21 (0; 1.08)	<0.0001
MHA	15 (15.0)	0.00 (0; 0.18)	27 (28.7)	1.28 (0.13; 5.06)	44 (24.3)	0.93 (0; 4.43)	

Irritability/nervousness							
HA	36 (36.0)	0.00 (0; 0.98)	35 (37.2)	0.28 (0.06; 1.38)	63 (34.8)	0.22 (0; 1.60)	<0.0001
MHA	36 (36.0)	0.00 (0; 0.31)	35 (37.2)	0.28 (0.06; 1.38)	63 (34.8)	1.18 (0; 7.46)^#^	

Memory loss							
HA	27 (27.0)	0.00 (0; 0.67)	29 (30.9)	0.27 (0.09; 1.05)	43 (23.8)	0.22 (0; 1.60)	<0.0001
MHA	27 (27.0)	0.00 (0; 0.31)	29 (30.9)	1.28 (0.22; 4.95)	43 (23.8)	1.26 (0; 7.46)	

Tingling							
HA	19 (19.0)	0.00 (0; 0.98)	28 (29.8)	0.30 (0.04; 1.38)	36 (19.9)	0.19 (0.02; 0.91)	<0.0001
MHA	19 (19.0)	0.00 (0; 0.11)	28 (29.8)	1.21 (0.16; 6.19)	36 (19.9)	1.14 (0.19; 5.50)	

Tremor							
HA	05 (5.0)	0.00 (0; 0.19)	09 (9.6)	0.25 (0.12; 1.20)	24 (13.3)	0.22 (0; 1.60)	0.0153
MHA	05 (5.0)	0.00 (0; 0)	09 (9.6)	1.25 (0.43; 5.29)	24 (13.3)	0.98 (0; 7.46)	0.0024

Weakness							
HA	12 (12.0)	0.00 (0; 0.43)	26 (27.7)^*∗*^	0.32 (0.06; 0.83)	32 (17.7)^*∗*^	0.25 (0; 1.60)	<0.0001
MHA	12 (12.0)	0.00 (0; 0.18)	26 (27.7)^*∗*^	1.24 (0.29; 3.74)	32 (17.7)^*∗*^	1.30 (0; 7.46)	

Weight loss							
HA	07 (7.0)	0.00 (0; 0.06)	21 (22.8)^*∗*^	0.25 (0; 0.91)	13 (7.2)	0.37 (0.12; 0.89)	<0.0001
MHA	07 (7.0)	0.00 (0; 0.12)	21 (22.8)^*∗*^	1.02 (0; 4.64)	13 (7.2)	1.88 (0.57; 3.62)	

^1^Not occupationally exposed to toluene and xylene. ^2^Occupationally exposed to toluene and xylene. ^3^Kruskal–Wallis test. ^*∗*^Higher frequency of HA and MHA biomarkers compared to OW or between exposed groups, CSW and FSA (chi-square test, *p* < 0.05). ^#^Higher level of HA and MHA biomarkers when CSW and FSA were compared (Kruskal–Wallis test with Dunn's posttest, *p* < 0.05).

## Data Availability

The data that support the findings of this study are available upon request from the corresponding author. The data are not publicly available due to containing information that could compromise the privacy of research participants.
